# Impact of Different Pasteurization Techniques and Subsequent Ultrasonication on the In Vitro Bioaccessibility of Carotenoids in Valencia Orange (*Citrus sinensis* (L.) Osbeck) Juice

**DOI:** 10.3390/antiox9060534

**Published:** 2020-06-18

**Authors:** Lara Etzbach, Ruth Stolle, Kerstin Anheuser, Volker Herdegen, Andreas Schieber, Fabian Weber

**Affiliations:** 1 Institute of Nutritional and Food Sciences, Molecular Food Technology, University of Bonn, Endenicher Allee 19b, D-53115 Bonn, Germany; lara.etzbach@uni-bonn.de (L.E.); s7rustol@uni-bonn.de (R.S.); schieber@uni-bonn.de (A.S.); 2 Eckes-Granini Group GmbH, Ludwig-Eckes-Platz 1, D-55268 Nieder-Olm, Germany; Kerstin.Anheuser@eckes-granini.com (K.A.); Volker.Herdegen@eckes-granini.com (V.H.)

**Keywords:** orange juice, *Citrus sinensis* L., carotenoids, in vitro bioaccessibility, pasteurization, ultrasonication, pulsed electric fields, high pressure processing

## Abstract

The effects of traditional pasteurization (low pasteurization, conventional pasteurization, hot filling) and alternative pasteurization (pulsed electric fields, high pressure processing), followed by ultrasonication on the carotenoid content, carotenoid profile, and on the in vitro carotenoid bioaccessibility of orange juice were investigated. There was no significant difference in the total carotenoid content between the untreated juice (879.74 µg/100 g juice) and all pasteurized juices. Significantly lower contents of violaxanthin esters were found in the high thermally-treated juices (conventional pasteurization, hot filling) compared to the untreated juice, owing to heat-induced epoxy-furanoid rearrangement. The additional ultrasonication had almost no effects on the carotenoid content and profile of the orange juices. However, the in vitro solubilization and the micellarization efficiency were strongly increased by ultrasound, the latter by approximately 85.3–159.5%. Therefore, among the applied processing techniques, ultrasonication might be a promising technology to enhance the in vitro bioaccessibility of carotenoids and, thus, the nutritional value of orange juice.

## 1. Introduction

Orange juice is the most popular fruit beverage and accounts for 60% of the consumption of fruit juices and juice-based drinks in Western Europe and North America [1,2]. Its popularity is at least partially based on the content of vitamin C, flavonoids, carotenoids, minerals, and fiber [3]. Scientific interest in carotenoids has grown since they demonstrated antioxidant properties [4], besides their coloring features, resulting in several health benefits [5,6].

Conventional thermal pasteurization is used to extend the shelf life of fruit juices and ensures stable and safe products. While necessary for the inactivation of microorganisms and enzymes, conventional thermal pasteurization might lead to detrimental changes in flavor, color, and in the content of bioactive compounds, such as carotenoids, since these compounds are susceptible to isomerization, oxidation, and cleavage initiated by heat, free radicals, oxygen, acids, and light [7,8]. Over the last few decades, non-thermal pasteurization techniques, such as high pressure processing (HPP) and pulsed electric fields (PEF), have been investigated to avoid such quality deterioration [7,9,10].

Ultrasonication has been shown to be a promising technology to increase the nutritional value of food juices and purees due to an enhanced disintegration of cell walls and membranes compensating the loss of bioactive compounds by food processing [11,12,13]. Since several studies showed that ultrasonication alone is usually not severe enough for complete microorganism and enzyme inactivation, the combination of ultrasonication with other preservation techniques is recommended [12,14].

Besides affecting the absolute content of carotenoids, owing to degradation and liberation, processing might enhance carotenoid bioavailability, due to the disaggregation of cell clusters and disruption of cell structures. Since the determination of the bioavailability in vivo is laborious, resource and time intensive, and could raise ethical issues, simple, inexpensive, and rapid in vitro methods have been developed [15]. Simulated in vitro digestion allows for the estimation of carotenoid bioaccessibility, that is, the amount of carotenoids released from the food matrix and incorporated into micelles and, thus, potentially available for intestinal absorption [16]. The absorption of carotenoids involves their liberation from the food matrix into the lipid portion of the stomach (solubilization), the incorporation into lipid droplets, the incorporation into mixed micelles with the help of bile acids and pancreatic enzymes in the duodenum, uptake by the enterocytes via passive diffusion or scavenger receptors, incorporation into chylomicrons, entering the lymphatic system, and circulation [15,17].

The bioaccessibility of bioactive compounds is of greater interest for the determination of the potential health-related properties of a juice than just the concentration in the juice, and these properties are substantially influenced by the processing technology [18]. Since relevant studies are not available, the aim of the present study was to assess the combined impact of pasteurization and ultrasonication on several important bioactive properties of orange juice.

The objective of the present study was to evaluate the impact of commonly applied pasteurization techniques (low pasteurization (LP), conventional pasteurization (CP), hot filling), and alternative approaches, such as pulsed electric fields (PEF) or high pressure processing (HPP) both with and without additional ultrasonication on the carotenoid content, carotenoid profile, in vitro carotenoid liberation, and in vitro carotenoid bioaccessibility of orange juice.

## 2. Materials and Methods

### 2.1. Orange Juice

Valencia oranges (*Citrus sinensis* (L.) Osbeck) were imported from South Africa. The juice was produced by Eckes-Granini Group GmbH (Nieder-Olm, Germany) using a citrus juice extractor (JBT Foodtech, Parma, Italy) and a perforated plate for homogenization. The homogenized juice was degassed. The processing parameters were based on microbial and enzymatic inactivation results to produce customary orange juices, and were assessed in preliminary tests by Eckes-Granini Group GmbH (Nieder-Olm, Germany).

### 2.2. Chemicals and Standards

Ultrapure water was obtained from a PURELAB flex 2 water purification system (ELGA LabWater, Paris, France). For the simulated gastrointestinal digestion, calcium chloride dihydrate and pyrogallol, both from Carl Roth GmbH & Co. KG (Karlsruhe, Germany), potassium chloride (Gruessing GmbH, Filsum, Germany), potassium dihydrogen phosphate (Merck, Darmstadt, Germany), sodium chloride (neoFroxx GmbH, Einhausen, Germany), magnesium chloride hexahydrate, sodium hydrogen carbonate, and hydrochloric acid 37%, all from VWR (Mannheim, Germany), were used. Enzymes and bile salts were purchased from Sigma-Aldrich (Munich, Germany)—lipase from *Aspergillus niger* (165.4 I.U./g), pancreatin from porcine pancreas (digestion power ≥3× USP specifications), pepsin from porcine stomach mucosa (63 I.U./mg solid), and porcine bile extract. Carotenoids were analyzed using acetone 99.8%, hexane (HPLC grade), and methanol, all from VWR (Mannheim, Germany), ethanol ≥99.8% (Sigma-Aldrich, Munich, Germany), and methyl *tert*-butyl ether (MTBE, HPLC grade) and butylated hydroxytoluene (BHT, ≥99.8%), both from Carl Roth GmbH & Co. KG (Karlsruhe, Germany). The β-carotene standard (≥97%) was from Sigma-Aldrich (Munich, Germany). Sodium acetate trihydrate (Bernd Kraft GmbH, Duisburg, Germany), oxalic acid dihydrate (Bernd Kraft GmbH, Duisburg, Germany), 1,2-dichloro-6-indophenol disodium salt (Merck Chemicals GmbH, Darmstadt, Germany), and an ascorbic acid standard (Honeywell GmbH, Seelze, Germany) were used for the estimation of ascorbic acid.

### 2.3. Thermal Pasteurization Treatments

Three different thermal pasteurization treatments (low pasteurization, conventional pasteurization, and hot filling) were applied. All thermal treatments were conducted at Eckes-Granini (Nieder-Olm, Germany) with a HTST (high-temperature-short-time)-system HT320 HTST/UHT (OMVE Netherlands B.V., Utrecht, Netherlands) at a flow rate of 90 L/h. Different pasteurization temperatures were used for the low and the conventional pasteurization at similar holding times of 30 s and 31 s, respectively, as shown in Table 1. In the case of hot filling, the juice was processed as conventionally pasteurized juice but was filled hot into glass bottles and transported to a cooling tunnel. Cold aseptic filling into polyethylene terephthalate (PET) bottles was performed for the low and conventional pasteurized juices. The juices were stored frozen at −20 °C until the simulated gastrointestinal digestion or additional ultrasonication.

### 2.4. Pulsed Electric Field Treatment

Pulsed electric field treatment was performed at a continuous flow pilot scale unit (ELEA^®^ HVP-5, Quakenbrueck, Germany). The pilot scale unit consists of two collinear treatment chambers, each with two titanium electrodes with a 10 mm electrogap connected to a liquid handling system with two heat exchangers and a filling unit. A pump was connected to the system, providing a flow rate of 42 L/h. For the pulsed electric field treatment, an electric field strength of 12.7 kV/cm, an energy input of 107.4 kJ/L, and a pulse frequency of 61 Hz were applied to the orange juice with inlet and outlet temperatures of 40.4 °C and 62.9 °C, respectively, as shown in Table 1. After the pulsed electric field treatment, the juice was immediately cooled to 5 °C and filled into PET-bottles.

### 2.5. High Pressure Processing

High pressure processing was carried out using an industrial Wave 6000/55 unit (55 L, 20 cm inner diameter, Hiperbaric, Burgos, Spain) at the German Institute of Food Technologies (DIL, Quakenbrueck, Germany). Pressurization was performed with tap water. The untreated juice was filled into PET bottles and the juice was subjected to 600 MPa for 3 min. Since the increase in the temperature, owing to compression, could not be measured during the high pressure processing, the temperature increase was estimated based on the literature data, as shown in Table 1 [19].

### 2.6. Ultrasound Treatment

For the additional ultrasonication, orange juice samples were thawed using a water bath (<25 °C), weighed into centrifuge tubes (30.0 ± 0.1 g) that were placed in a beaker containing ice water. A UP200St ultrasonic processor (200 W, 26 kHz) with a S26d7 titanium probe of 38.5 mm^2^ front surface with a diameter of 7 mm (Hielscher Ultrasonics, Teltow, Germany) was used. The ultrasonic probe was immersed 1 cm for sonication. The samples were ultrasonicated at an amplitude of 80% for 12 min with a pulse frequency of 50%. Table 2 shows the powers, intensities, and temperatures during the ultrasonication treatments. After sonication, the samples were stored frozen at −20 °C until carotenoid extraction or in vitro gastrointestinal digestion.

### 2.7. Estimation of Ascorbic Acid Content

The ascorbic acid concentration was titrimetrically determined in triplicate using 1,2-dichloro-6-indophenol as an indicator, as described previously [20]. Two milliliters of the orange juice were dissolved with 5 mL of 10% (*v/v*) sodium acetate and made up to 80 mL with 2% (*v/v*) oxalic acid. The mixture was titrated until the color changed from blue to pink. A blank and an ascorbic acid standard were used to define the titer of the indicator.

### 2.8. Simulated In Vitro Gastrointestional Model and Bioaccessibility of Carotenoids

Both the untreated and the pasteurized juices with and without additional ultrasonication were subjected to a simulated digestion described by several studies [21,22] with slight modifications. The in vitro digestion applied in the present study consisted of a simulated gastric and small intestine phase, since the oral phase is negligible in the case of fruit juices.

For the simulated digestion, juices were thawed in a water bath (<25 °C) and weighed (4.00 ± 0.02 g) into centrifuge tubes. The gastric phase was initiated by the addition of a 1.5 mL electrolyte solution (3 g/L NaCl, 1.1 g/L KCl, 1.5 g/L CaCl_2_·2 H_2_O, 0.87 g/L KH_2_PO_4_, 0.7 g/L MgCl_2_·6 H_2_O) and 2 mL of 0.5 mg/mL porcine pepsin (63 I.U./mg solid) in electrolyte solution. The mixture was vortexed for 1 min and the pH was adjusted to 2.5 using 1 M HCl. The centrifuge tubes were flushed with nitrogen and the samples were incubated in a shaking water bath at 100 rpm and 37 °C for 1 h.

After the pH of the small intestine phase was adjusted to 6.9 using a 1 M NaHCO_3_ solution, 1.5 mL of a bile salt/enzyme solution (8 g/L pancreatin, 30 g/L porcine bile salts, 3 g/L lipase, 5 g/L pyrogallol in ultrapure water) was added. The samples were vortexed, flushed again with nitrogen, and incubated at 37 °C and 100 rpm for 2 h.

After the simulated digestion, the samples were made up to 10 mL with ultrapure water and aliquots (2.5 mL) were taken for the total fraction. The residual sample was centrifuged with a Heraeus Megafuge 40R Centrifuge (Thermo Fisher Scientific, Braunschweig, Germany) for 45 min at 5 °C and 18,500 g. An aliquot (3 mL) of the supernatant containing the liberated carotenoids from the fruit pulp was taken (soluble fraction) and, in addition, aliquots (3 mL) of the micellar fraction were obtained after filtering the supernatant through 0.2 μm Chromafil RC-20/15 MS filters (Macherey-Nagel, Dueren, Germany). All fractions were stored at −20 °C until carotenoid extraction.

The micellarization efficiency was calculated as the percentage of carotenoid content that was incorporated into the micelles using the following equation:

(1) $$\mathrm{micellarization}\;\mathrm{efficiency}\;\left(\%\right)=\frac{\mathrm{carotenoid}\;\mathrm{content}\;\mathrm{in}\;\mathrm{the}\;\mathrm{micellar}\;\mathrm{fraction}}{\mathrm{mean}\;\mathrm{carotenoid}\;\mathrm{content}\;\mathrm{in}\;\mathrm{the}\;\mathrm{orange}\;\mathrm{juice}}\times 100$$

### 2.9. Carotenoid Analysis

#### 2.9.1. Carotenoid Extraction

Carotenoids were analyzed in triplicate in orange juices and digestion fractions (total, soluble, and micellar fraction). The orange juices were extracted by weighing 4.00 ± 0.02 g in centrifuge tubes, according to the previously published method [23]. In the case of the digestion fractions, the extraction step was repeated twice instead of three times because of lower carotenoid concentrations compared to the non-digested orange juices. The extracts were evaporated to dryness with nitrogen and stored at −80 °C until analysis. One sample was saponified to ease the identification of orange juice carotenoids [24].

For HPLC(-MS) analysis, the evaporated extracts of the orange juices and digestion fractions were made up to 1000 and 600 µL, respectively, with methanol/methyl *tert*-butyl ether (50:50, *v/v*) containing 0.1% BHT, filtered through 0.2 μm Chromafil RC-20/15 MS filters (Macherey-Nagel Dueren, Germany), and used for HPLC-DAD(-MS) analysis.

#### 2.9.2. Quantification and Identification of Carotenoids

The carotenoids were quantified using a Prominence UFLC system (Shimadzu, Kyoto, Japan), equipped with two Nexera X2 LC-30AD high-pressure gradient pumps, a Prominence DGU-20A5R degasser, a Nexera SIL-30AC Prominence autosampler (15 °C, injection volume 10 and 20 µL for orange juices and digestion fractions, respectively), a CTO-20AC Prominence column oven at 25 °C, and a SPD-M20A Prominence diode array detector. The carotenoids were separated on a C30 Accucore column (2.6 µm, 150 × 2.1 mm) (Thermo Fisher Scientific, Braunschweig, Germany) using methanol/methyl *tert*-butyl ether/water as eluent A (85/5/10, *v/v/v*) and as eluent B (11/85/4, *v/v/v*). The gradient program (flow rate of 0.4 mL/min) was as follows: 0 min, 2% B; 7 min, 2% B; 20 min, 23% B; 52.5 min, 71% B; 53.5 min, 100% B; 55 min, 100% B; 56.5 min, 2% B; 60 min, 2% B. The total carotenoid content was calculated as the sum of the individual carotenoids. The carotenoids were quantified as β-carotene equivalents using an external calibration curve at 450 nm. Data acquisition was performed by the LabSolutions software, version 5.85 (Shimadzu, Kyoto, Japan).

For carotenoid identification, the samples were analyzed on an Acquity UPLC I-Class system (Waters, Eschborn, Germany), consisting of a binary solvent manager, a sample Manager-FL at 15 °C, a column oven at 25 °C, and a PDA eλ Detector. The UPLC was coupled with a LTQ-XL ion trap mass spectrometer equipped with an APCI ionization source (Thermo Fisher Scientific, Braunschweig, Germany), operating in positive ion mode in a mass range of *m/z* 200–2000. The applied chromatographic conditions (temperatures, column, gradient program, flow rate, and injection volume) were the same as mentioned before. Ammonium formate (5 mmol/L) was added to the eluents to improve ionization. The following MS parameters were used: collision gas, helium; capillary temperature, 275 °C; APCI vaporizer temperature, 400 °C; sheath gas flow, 30 arbitrary units; aux gas flow, 5 arbitrary units; sweep gas flow, 5 arbitrary units; source voltage, 6 kV; source current, 5 µA; capillary voltage, 15 V; tube lens, 65 V. MS data acquisition was performed by the Xcalibur 2.2 SP1.48 software from Thermo Fisher Scientific (Braunschweig, Germany).

The carotenoids were identified based on their retention time, elution order on the Accucore C30 column, UV/Vis spectrum (positions of absorption maxima λ_max_ and spectral fine structure expressed as %III/II), and mass spectrum compared to the data available in the literature [25,26,27,28].

### 2.10. Statistical Analysis

Statistical analysis was carried out using the XLSTAT software, version 2014.4.06 (Addinsoft, Paris, France). An ANOVA with Bonferroni post-hoc test was performed to determine significant differences (*p* ≤ 0.05).

## 3. Results and Discussion

### 3.1. Ascorbic Acid Content

The ascorbic acid content was significantly decreased by about 3–5% after all pasteurization techniques, compared to the untreated juice, as shown in Table 1, but the losses are within the range of previous studies for conventional and alternative pasteurization [29,30]. Despite the thermal sensitivity of ascorbic acid, the losses are relatively low because of the stabilizing effects of the low pH value. The significant differences between the pasteurization techniques might not be relevant regarding the final product from a consumer perspective, since the standard deviation is very small.

### 3.2. Carotenoid Identification

In our study, 92 carotenoids were detected in orange juice by HPLC-DAD-APCI-MS*^n^*. The chromatograms of orange juice before and after in vitro digestion are shown in Figure S1. Peak identification and characterization are presented in Table 3. From the 92 carotenoids, 61 carotenoids were tentatively identified. In addition, the parent carotenoids of 16 unknown carotenoid esters were tentatively identified with the help of the UV/Vis spectrum and the *m/z* of the carotenoid.

The carotenoid pattern of orange juice is one of the most complex among fruits and is dominated by carotenoid fatty acid esters, as previously reported [26,31,32,33]. The main carotenoids in orange juice are esters of violaxanthin, β-cryptoxanthin, antheraxanthin, and mutatoxanthin esterified mostly with myristic acid (C_14:0_), palmitic acid (C_16:0_), and lauric acid (C_12:0_) as monoesters or diesters. The carotenoid profile of orange juice reveals only small amounts of free xanthophylls and carotenes, such as α-carotene, β-carotene, and ζ-carotene.

Because of the natural acidity of the juice, the carotenoid pattern of orange juice is dominated by epoxy carotenoid esters and the rearrangement of 5,6-epoxy carotenoids, such as violaxanthin and antheraxanthin, to their respective 5,8-epoxy carotenoids (luteoxanthin, mutatoxanthin, auroxanthin) is favored [34,35,36]. In the present study, the identification of (*Z*)-isomers was difficult, owing to a poor definition of the (*Z*)-peak, but the peaks were assigned due to hypsochromic shifting in λ_max_, a reduced spectral fine structure and elution order. Because of the susceptibility of epoxides toward isomerization, high contents of the (*Z*)-isomers of several epoxy carotenoid fatty acid esters, such as violaxanthin esters, were found in orange juice, as previously reported [26,32,33].

Interestingly, apart from the common C_40_ carotenoids and their esters, an 8′-apocarotenoid (peak 2a, *m/z* 435, C_30_H_42_O_2_) was detected in the present study. Since the fragment ion at *m/z* 417 was only observed in small intensities (8%), the loss of water [M + H - 18]^+^ is assumed to be attributed to the loss of an epoxy group instead of a hydroxy group. The apocarotenoid could be a cleavage product of the epoxy carotenoids native in orange juice. Since the apocarotenoid was found in the non-digested samples in small amounts only, the formation of the apocarotenoid might be enhanced by the conditions during the gastric and small intestine phase by non-enzymatic oxidation processes via reactive oxygen species or autoxidation [37,38]. Little is known about the in vitro and in vivo absorption of apocarotenoids formed by non-enzymatic and oxidative cleavage. Several apocarotenals, apolycopenals, and β-apo-13-carotenone, primarily resulting from the enzymatic cleavage of carotenoids, were found in vitro and in vivo [39,40,41].

### 3.3. Effects of Pasteurization on the Carotenoid Content and Profile

**Figure 1:**
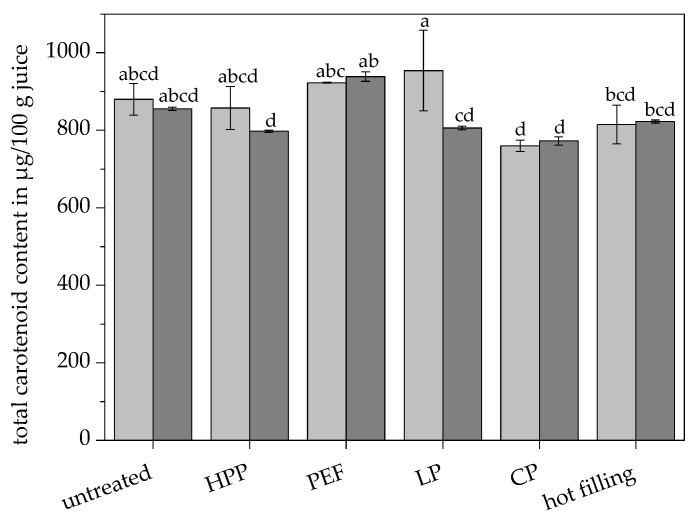
Total carotenoid contents in the untreated and pasteurized juices without (■) and with (■) ultrasonication. Different letters indicate significant differences (*p* ≤ 0.05). Values are means ± standard deviation (*n* = 3).

No significant differences in the total carotenoid content were observed between the untreated juice (879.74 µg/100 g juice) and all pasteurized juices, shown in Figure 1.

Contradictory observations concerning the effects of different pasteurization techniques on the carotenoid content in orange juice are stated in the literature, which can be attributed to different processing conditions, such as juice extraction, pasteurization conditions, and equipment. Several studies observed no significant differences compared to untreated orange juice in the case of low thermal pasteurization [7], high pressure processing [42,43,44], pulsed electric field technology [7,29,42,44], and traditional pasteurization [29]. Significantly higher carotenoid contents in orange juice treated with high pressure were shown by Plaza et al. [7] and Sánchez-Moreno et al. [10,29], whereas some studies described a reduced carotenoid content in thermally-treated orange juices [3,45,46]. Depending on the strength of the pulsed electric field treatment, increased and decreased carotenoid contents were both described [46]. In the present study, the conventionally pasteurized juice (759.95 µg/100 g juice) and the hot filled juice (815.30 µg/100 g juice) showed a significantly lower total carotenoid content compared to the low pasteurized juice (954.10 µg/100 g juice), as shown in Figure 1. High temperatures during processing, such as conventional pasteurization and hot filling might cause the instability of the polyene chain of the carotenoids, resulting in carotenoid degradation trough isomerization, oxidation, and cleavage. Overall, the differences are rather low and the observed losses might not be of high relevance for the final product regarding the consumer’s point of view.

**Figure 2:**
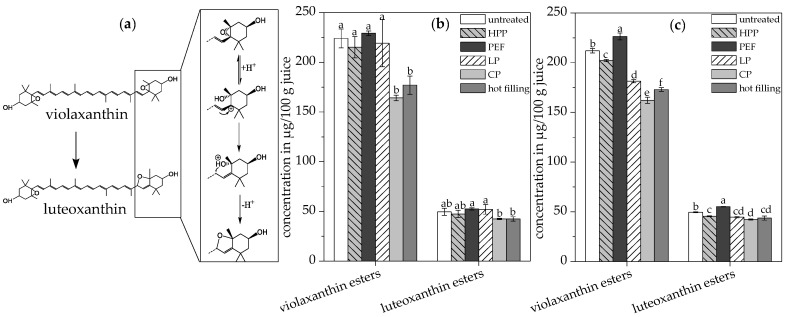
(**a**) Rearrangement of violaxanthin to luteoxanthin according to Schieber et al. [35]. Violaxanthin and luteoxanthin ester contents in the untreated and pasteurized juices without (**b**) and with (**c**) ultrasonication. Different letters indicate significant differences (*p* ≤ 0.05). Values are means ± standard deviation (*n* = 3).

The carotenoid profile of orange juice was affected by the different pasteurization techniques. Conventional pasteurization and hot filling led to a significantly lower content of total violaxanthin esters of about 20.9% and 26.7%, respectively, compared to the untreated juice. The low pasteurized juice and both alternatively pasteurized juices showed no significant differences in the total violaxanthin ester content compared to the untreated juice, as shown in Table 4 and Figure 2b.

In the cases of violaxanthin laureate–myristate (peak 56) and violaxanthin dimyristate (peak 60), the pasteurization techniques were classified in three significance groups of high thermal pasteurization (hot filling, conventional pasteurization), low pasteurization, and alternative/no pasteurization (PEF, HPP, untreated), due to significantly increasing contents, respectively, as shown in Table 4. Non-epoxy carotenoid esters, such as lutein, zeaxanthin, zeinoxanthin, and β-cryptoxanthin esters were hardly affected by any pasteurization technique.

Changes in the content of provitamin A active carotenoids (β-cryptoxanthin, α-carotene, and β-carotene), free monohydroxylated, and polyhydroxylated xanthophylls were not significant for all pasteurization techniques, as shown in Table 4. The higher stability of carotenes and monohydroxylated xanthophylls compared to polyhydroxylated xanthophylls is well known [47,48,49]. The degradation of mono- and polyhydroxylated xanthophylls is probably compensated by the release of free xanthophylls due to the thermally induced hydrolysis of fatty acids from the carotenoid backbone.

Despite the stabilizing effects of fatty acids on carotenoids, epoxy carotenoid fatty acid esters, especially those esterified with violaxanthin, showed very high susceptibility toward thermally induced degradation. The total content of the diepoxy carotenoids was significantly lower by about 24.5 and 19.8% in the conventionally pasteurized juice and hot filled juice, respectively, compared to the untreated juice (273.52 µg/100 g juice). In contrast, the content of the monoepoxides showed no significant difference in all pasteurized juices, compared to the untreated juice.

Violaxanthin, an 5,6,5′,6′-diepoxycarotenoid, is considered one of the most labile carotenoids because it is easily isomerized in the presence of acids to its furanoid isomers luteoxanthin (5,6,5′,8′-diepoxycarotenoid), as shown in Figure 2a, and auroxanthin (5,8,5′,8′-diepoxycarotenoid) [49]. No significant differences in the mutatoxanthin (5,8-epoxycarotenoid) and luteoxanthin ester content were found between the untreated juice and all pasteurized juices. Interestingly, the detected violaxanthin esters are mainly diesters, whereas the luteoxanthin esters are primarily monoesters. It is probable that the contents of the 5,8-epoxy carotenoids are affected by the simultaneous formation and degradation of the compounds concomitant with the partial release of the fatty acids from the carotenoid backbone. Auroxanthin was not detected in the juices before in vitro digestion. Besides juice acidity [34], rearrangement from the 5,6- to 5,8-epoxides is enhanced by temperature and, therefore, affected by the processing technique. Hence, the rearrangement of epoxy groups could be employed to assess the heat exposure of orange juices, but this should be done carefully since rearrangements might take place slowly and also during the shelf life of the juice [34,46].

### 3.4. Effects of Additional Ultrasonication on the Carotenoid Content and Profile of Pasteurized Juices

Additional ultrasonication showed no effects on the total carotenoid content in the case of the untreated, HPP-treated, PEF-treated, conventional pasteurized, and hot filled juice, as shown in Figure 1. The total carotenoid content of the low pasteurized juice was significantly reduced by 16% due to ultrasonication. Free radicals produced by cavitation might be involved in the degradation of the carotenoids [50]. All pasteurization techniques, except for the PEF treatment, showed a significantly lower total carotenoid content in combination with ultrasonication, compared to the untreated juice. This loss can be attributed to carotenoid degradation caused by cavitation and high temperatures or high pressure, respectively.

Besides the highest significant total carotenoid content of 938.46 µg/100 g, the PEF-treated juice showed the highest significant content of total epoxy carotenoids, violaxanthin esters, total carotenoid esters, carotenes, diepoxy carotenoids, monoepoxy carotenoids, and free monohydroxylated xanthophylls after ultrasonication, compared to all other juices, as shown in Table 5. The combination of three different destructive effects (electroporation, cavitation, and temperature) on the cell walls led to the highest extractability of carotenoids. Electroporation causes the permeabilization of the cell membrane by the external electric field [51]. Hence, cavitation led to an enhanced release of carotenoids due to the disintegration of cells and chromoplasts, which might have been weakened before by the electro-permeabilizing effects of the PEF treatment and might, therefore, compensate carotenoid losses, owing to ultrasonication.

Ultrasonication had only minor effects on the absolute content of total violaxanthin and luteoxanthin esters in the juices, as shown in Figure 2c. Hence, the rearrangement of the 5,6,5′,6′-diepoxycarotenoid violaxanthin is not enhanced by the ultrasound treatment applied in the present study (T < 38 °C). Free radicals could promote both (*Z*)-isomerization reactions through the formation of carotenoid diradicals, called Doering’s diradicals, and oxidation processes through radical species produced by ultrasound action with water [52,53]. Since most of the carotenoids in orange juice are already epoxides, the free radicals formed through ultrasonication did not lead to significant carotenoid oxidation. In the present study, only slightly higher (*Z*)-isomerization in the ultrasonicated samples was observed.

### 3.5. Effects of Pasteurization on the In Vitro Bioaccesbility of Orange Juice Carotenoids

**Figure 3:**
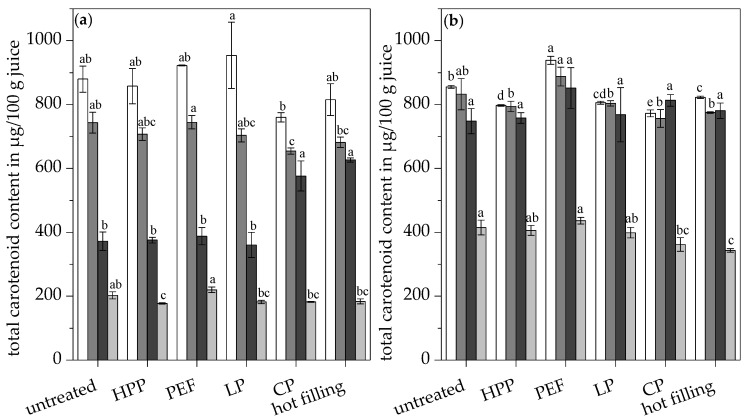
Total carotenoid concentration in the juices (□) and in the total fraction (■), soluble fraction (■), and micellar fraction (■) of the untreated and pasteurized juices without (**a**) and with (**b**) ultrasonication. Different letters indicate significant differences (*p* ≤ 0.05). Values are means ± standard deviation (*n* = 3).

After the simulated digestion, all of the digested samples were separated in three fractions: the total fraction containing all carotenoids in the sample after digestion, the soluble fraction, and the micellar fraction. Carotenoids are only bioaccessible when they are micellarized during digestion, rendering them potentially bioavailable in humans. The in vitro digestion model led to a reduction in the total carotenoid content in the total fraction of about 13.9–25.6%, depending on the pasteurization technique, as shown in Figure 3a. Several carotenoids (peaks 1, 2, 5, 6, 7, 10, 11, 12, 13, 14, and 22) were degraded. However, the formation of other carotenoids was observed (peaks 1a, 1b, 1c, 2a, 11a, 11b, 13a und 13b), as shown in Figure S1.

The detrimental conditions during the in vitro digestion, such as the low pH value and the elevated temperature of 37 °C for 3 h, resulted in the degradation of carotenoids due to oxidation, isomerization, and cleavage processes. (All-*E*)-mutatoxanthin and its (*Z*)-mutatoxanthin isomer I were not detected in the digested samples, whereas a newly formed (*Z*)-mutatoxanthin isomer II and (all-*E*)-auroxanthin, the di-5,8-epoxide of mutatoxanthin, were found. The ratio of luteoxanthin esters to violaxanthin esters was not shifted toward the luteoxanthin esters, which was assumed to be due to the degradation of violaxanthin, concomitant with the formation of luteoxanthin because of acid-catalyzed epoxy-furanoid rearrangement, caused by the low pH value of the gastric phase [54]. However, the contents of both carotenoid groups decreased after in vitro digestion, as shown in Table 4, Table 5 and Table 6.

**Figure 4:**
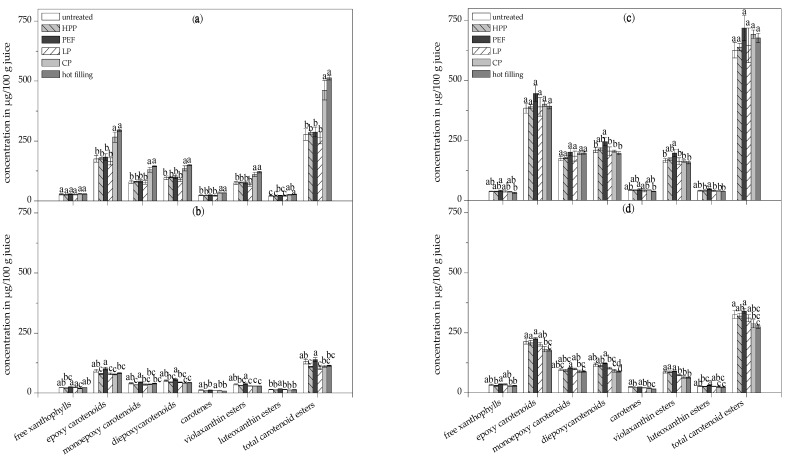
Concentrations of several carotenoid groups in the soluble (**a**) and micellar fractions (**b**) of the untreated and pasteurized orange juices without ultrasonication, and in the soluble (**c**) and micellar fractions (**d**) of the ultrasonicated juices. Different letters indicate significant differences (*p* ≤ 0.05). Values are means ± standard deviation (*n* = 3).

The lowest total carotenoid contents in the total fraction (insoluble and soluble carotenoids) were observed in the high thermally-treated juices (hot filling and conventional pasteurization), as shown in Figure 3a, due to preceding carotenoid degradation to non-colored cleavage products by the high temperatures. Nevertheless, the total fraction of the conventionally pasteurized and hot filled juice contained the significantly highest proportions of soluble carotenoids of 88.1 and 90.7%, respectively, compared to the other juices (<53.2%), as shown in Figure 3a. Similar effects were observed in the carotenoid profile for epoxy carotenoids, monoepoxy carotenoids, diepoxy carotenoids, carotenes, total carotenoid esters, and violaxanthin esters, showing significantly higher concentrations in the soluble fraction of the hot filled and conventionally pasteurized juice compared to the untreated juice and all other pasteurized juices, as shown in Figure 4a.

The incorporation of carotenoids of low polarity, such as carotenoid fatty acid esters into lipid droplets during the gastric phase (solubilization), is restricted, since these carotenoids have to reach the core of the lipid droplets. Carotenes are located in the triacylglycerol-rich core of the lipid droplets, whereas the more polar xanthophylls are more likely found near the surface monolayer, together with proteins, phospholipids, and fatty acids [17]. The location strongly influences the incorporation of carotenoids into mixed micelles, since carotenoids near the lipid droplet surface are more accessible for micellarization.

No significant difference in the content of free xanthophylls was found in the soluble fraction of the juices, as shown in Figure 4a. This might be explained by the higher solubility of free xanthophylls compared to carotenes, which results in complete solubilization.

Despite the enhanced release of carotenoids from the fruit matrix and their incorporation into lipid droplets due to heat, the total carotenoid concentration in the micellar fraction of the hot filled and conventionally pasteurized juice was not significantly higher compared to the untreated and all other pasteurized juices, as shown in Figure 3a. Thus, the high thermal treatments just enhanced the solubilization of the carotenoids in the gastric phase, but could not increase the micellarization in the small intestine phase. Carotenoids liberated from the fruit matrix by heat in the case of the high thermally-treated juices might be in a structure not favored for micellarization, such as large lipid droplets, carotenoid aggregates, or crystals. The added bile salts, acting as surfactants, were not able to reduce the size of the formed large lipid globules. The structure and size of the lipid droplets emulsified in the juice play an important role in the incorporation of carotenoids into micelles.

**Figure 5:**
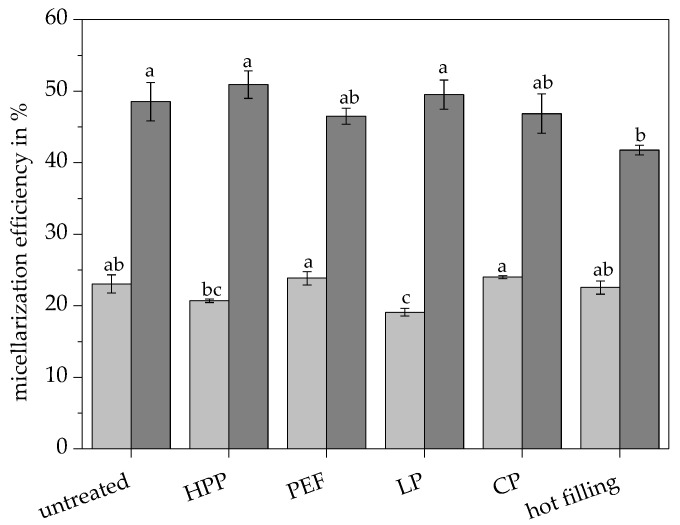
Carotenoid micellarization efficiencies in the untreated and pasteurized orange juices without (■) and with (■) additional ultrasonication. Different letters indicate significant differences (*p* ≤ 0.05). Values are means ± standard deviation (*n* = 3).

The highest total carotenoid concentration in the micellar fraction of 220.08 µg/100 g was found in the PEF-treated juice and was significantly higher compared to all other pasteurized juices, as shown in Figure 3a. The PEF-treated juice also showed significantly higher contents of total epoxy carotenoids, monoepoxy carotenoids, diepoxy carotenoids, total carotenoid fatty acid esters, carotenes, luteoxanthin esters, and violaxanthin esters in the micellar fraction compared to all other treated juices, as shown in Figure 4b. The micellar fraction of the conventionally pasteurized juices (low pasteurization, conventional pasteurization, and hot filling) exhibited significantly lower contents of violaxanthin esters and diepoxy carotenoids compared to the untreated and PEF-treated juice, owing to the high susceptibility of epoxy carotenoids toward thermal degradation [49]. Despite the high total carotenoid contents of the orange juices, only 19.1% to 24.0% of the carotenoids were transferred into mixed micelles, as shown in Figure 5.

Pasteurization techniques might enhance the bioaccessibility of carotenoids through matrix disruption by heat, pressure, or electroporation [55,56,57,58,59], but in the present study none of the applied pasteurization techniques significantly increased the micellarization efficiency of the carotenoids compared to the untreated juice, as shown in Figure 5. Rodríguez-Roque et al. [60] also reported an insufficiency of several pasteurization techniques, such as high-intensity pulsed electric fields, high-pressure processing, and thermal treatment, to increase the bioaccessibility of the carotenoids in a water based fruit juice beverage.

### 3.6. Effects of Additional Ultrasonication on the In Vitro Carotenoid Bioaccessbility of Pasteurized Orange Juices

Additional ultrasonication led to high carotenoid concentrations in the total fraction of all the juices after the in vitro digestion model, as shown in Figure 3b, owing to both the higher digestive stability of carotenoids and the enhanced cell and chromoplast disintegration. The total carotenoid content in the total fraction was reduced by about 0.2–5.8% by the in vitro digestion. Moreover, ultrasonication maximized the proportion of soluble carotenoids and, therefore, of potential bioaccessible carotenoids in all of the digested samples, regardless of the pasteurization technique due to the disruption of cell and chromoplast aggregates by cavitation, as shown in Figure 3b. Thus, carotenoid liberation was much higher with ultrasonication (>89.9%) in the present study than reported for orange juice in the literature (34–53.9%) [61,62]. There were no significant differences in the soluble fractions of all the ultrasonicated samples regarding total carotenoids, epoxy carotenoids, monoepoxy carotenoids, and carotenoid fatty acid esters, as shown in Figure 3b and Figure 4c.

Despite similar initial total carotenoid contents in the juices, the total carotenoid content in the micelles was significantly increased to 343.44–436.33 µg/100 g in the ultrasonicated samples compared to 177.41–220.08 µg/100 g in the non-ultrasonicated samples, as shown in Figure 3. Thus, the micellarization efficiency of total carotenoids was significantly increased by 85.3–159.5% to 41.7–50.9% by ultrasonication in the differently pasteurized juices, respectively, as shown in Figure 5. The low- and non-thermally-treated juices, such as the untreated, high pressure-treated, and low pasteurized juice, benefited most from the additional ultrasound treatment because of a lower degree of decomposition by the previously applied pasteurization technique. However, there was no significant difference in the carotenoid micellarization efficiency of the pasteurized ultrasonicated juices compared to the untreated ultrasonicated juice, except for the hot filled ultrasonicated juice, which showed a significantly reduced micellarization efficiency due to advanced carotenoid degradation, as shown in Figure 5.

The micellar fractions of the juices with high heat exposure (conventional pasteurization and hot filling) showed significantly lower contents in total carotenoids, epoxy carotenoids, diepoxy carotenoids, and carotenoid fatty acid esters compared to the untreated juice, owing to high temperatures leading to carotenoid degradation, as shown in Figure 3b and Figure 4c. Violaxanthin esters were significantly reduced in the micelles of all traditionally pasteurized juices (low pasteurization, conventional pasteurization, hot filling), but not in the alternatively pasteurized juices (HPP and PEF) compared to the untreated juice, as shown in Figure 4d. The conditions during in vitro digestion did not result in a shift in the ratio of luteoxanthin to violaxanthin esters toward the luteoxanthin esters in all ultrasonicated and non-ultrasonicated samples, as shown in Table 6. Thus, the rearrangement of violaxanthin is initiated by temperature rather than by ultrasound or by the acidic conditions during the in vitro digestion.

The formation, growth, and implosive collapse of cavitation gas bubbles near solid surfaces, such as fruit pulp, may lead to the formation of microjets [63]. Microjets might facilitate lipid emulsification by generating local turbulences and liquid micro-circulations and, therefore, reducing the size of lipid droplets. The size of lipid droplets strongly affects the micellarization of carotenoids, since surface area enlargement allows for a greater interaction between lipolytic enzymes, bile salts, and lipid droplets, improving lipolysis, lipid digestion, and the formation of micelles [64]. Since the amount of enzymes and bile salts, facilitating carotenoid bioaccessibility with increasing contents [65], was always the same in the present study, the higher micellarization efficiencies are probably caused by the reduced size of lipid droplets through ultrasonication. A higher bioaccessibility of carotenoids after ultrasonication was also reported for *Chlorella vulgaris* [66], *Phaeodactylum tricornutum* [67], guava juice [68], and a fruit juice mixture [69].

### 3.7. Micellarization Efficiency and Carotenoid Structure

**Figure 6:**
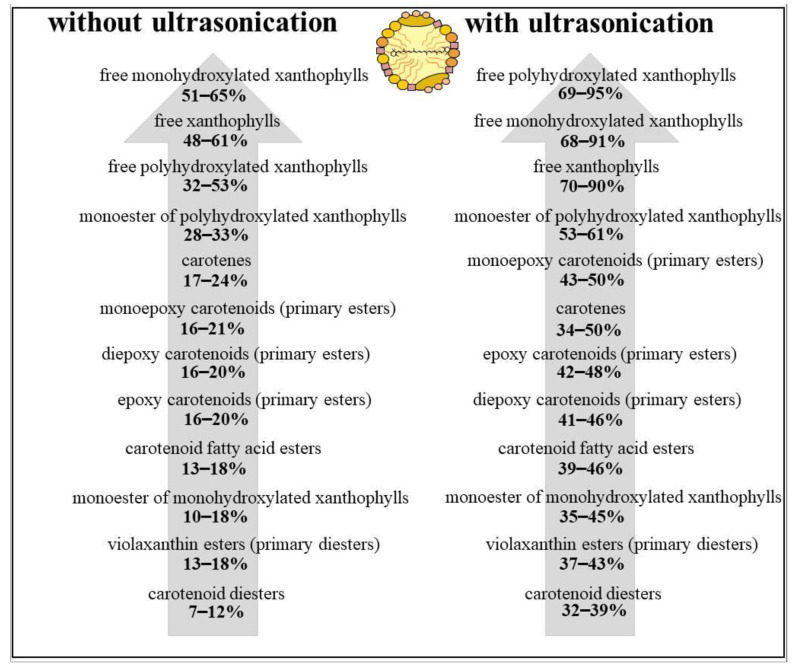
Micellarization efficiencies of several carotenoid groups in the orange juices without and with ultrasonication.

The inverse relationship between the micellarization efficiency and the hydrophobicity of carotenoids is well known [70,71,72], but studies with non-saponified and complex carotenoid profiles are scarce. The enhanced hydrophilicity of free xanthophylls compared to esterified xanthophylls and carotenes fostered the incorporation into micelles, as shown in Figure 6. Interestingly, the micellarization efficiency of monoesters of polyhydroxylated xanthophylls exceeded the incorporation of carotenes despite the fatty acid moiety. A free hydroxy group seems to promote the localization of the carotenoid at the outer layer of the lipid droplets and, thus, increases the incorporation into mixed micelles during the duodenal phase. Monoesters of monohydroxylated xanthophylls accordingly show lower micellarization efficiencies. Carotenoid fatty acid diesters showed the lowest micellarization efficiency, owing to the two fatty acid moieties increasing the hydrophobicity of the carotenoid.

With additional ultrasonication, the micellarization efficiency of free xanthophylls was increased from 48–61% to 70–90%, as shown in Figure 6. Moreover, ultrasonication raised the incorporation of carotenes into micelles from 17–24% to 34–50%, thus increasing the nutritional value of the juices through higher contents of potentially bioaccessible provitamin A active carotenoids. The micellarization efficiencies of the ultrasonicated juices are much higher than the values reported for orange juice, whereby the values of the non-ultrasonicated juices are within the range of previously published data [61,62]. It should be mentioned that almost all studies on the in vitro bioaccessibility of carotenoids in orange juice were carried out with saponification [16,61,62]. Total micellarization efficiencies, including free xanthophylls and carotenes, of 28.3% and 37.6–39.5% were found in the literature for fresh and pasteurized orange juices, respectively [62].

It should also be noted that carotenoid esters are effectively hydrolyzed in vivo during the absorption in the small intestine [73,74], while the satisfactory cleavage of carotenoid esters has not yet been achieved in vitro [70,75,76,77,78,79]. In the study of Granado-Lorencio et al. [75], the hydrolysis of carotenoid fatty acid esters was incomplete, regardless of the substrate-to-enzyme ratio, time of duodenal incubation, and the addition of other substances, such as colipase and phospholipase. The type of added enzymes during the small intestine phase (i.e., pancreatin, lipase, cholesteryl esterase) and the source of these enzymes (i.e., microorganisms, porcine, human) might affect the degree of carotenoid ester hydrolysis [75]. Besides the cleavage of carotenoid fatty acid esters in the duodenal fluid, micellized esters might be cleaved at the brush border membrane of the intestine or within the enterocyte. The brush border membrane of rats and humans were found to contain a pancreatic-derived esterase and an intrinsic esterase, which cleave retinyl esters [80].

However, in vitro models are suitable tools for the initial screening of bioaccessibility to investigate food-related factors, such as the processing technique on a variety of foods in a simple, inexpensive, and rapid way without any ethical issues [15].

## 4. Conclusions

Among the applied processing techniques, ultrasonication proved to be an effective tool to enhance the solubilization and micellarization of carotenoids in orange juice, concomitant with marginal effects on the carotenoid contents and profiles of the juices. Up to 159% higher micellarization was achieved by additional ultrasonication. High thermal pasteurization techniques, such as conventional pasteurization and hot filling, showed high epoxy-furanoid rearrangement of violaxanthin esters. Neither traditional (low pasteurization, conventional pasteurization, hot filling) nor alternative pasteurization approaches (pulsed electric fields and high pressure processing) significantly affected the micellarization efficiency of carotenoids compared to the untreated juice.

Ultrasound might facilitate the lipid emulsification and might reduce the size of lipid droplets during in vitro digestion, enhancing the incorporation of carotenoids into micelles. There is an increased need for research on the effects of ultrasound on the formation of lipid droplets during digestion. Since ultrasonication may affect the storage stability of carotenoids, in vitro bioaccessibility should be also assessed in stored samples. It still needs to be investigated to what extent ultrasonication may enhance the bioavailability of carotenoids in vivo, and whether the application of ultrasound on food systems may compensate a decreased bile acid secretion, which causes lipid and carotenoid malabsorption. Ultrasound technology represents a promising, environmentally friendly, and cost-saving technology for enhancing the nutritional value and provitamin A activity of juices by increasing the content of potentially bioaccessible carotenoids. However, not all of the effects induced by ultrasound in the food matrix have yet been revealed.

## Figures and Tables

**Table 1:** Product temperatures during the different process steps applied to Valencia orange juice and ascorbic acid contents. Different letters indicate significant differences (*p* ≤ 0.05). Ascorbic acid concentrations are means ± standard deviation (*n* = 3). HPP: high pressure processing; PEF: pulsed electric fields; LP: low pasteurization; CP: conventional pasteurization.

	Untreated	HPP	PEF	LP	CP	Hot Filling
Product inlet (°C)	-	10.5	-	8.1	9.0	9.5
Preheating (°C)	-	-	42.0	60.5	60.2	60.2
Before pasteurization (°C)	-	-	40.4	-	-	-
During pasteurization (°C)	-	24.5 ^1^	69.0 ^1^	73.9	92.2	92.2
Pasteurization end (°C)	-	12.0	62.9	72.5	89.6	89.6
Cooling (°C)	-	-	5.0	30.1	33.4	-
Filling (°C)	-	-	6.8	13.9	14.0	82.3
After cooling tunnel (°C)	-	-	-	-	-	37.2
Ascorbic acid (mg/L)	607.0 ± 3.6 ^a^	575.0 ± 1.4 ^d^	581.0 ± 1.4 ^cd^	583.7 ± 2.4 ^bc^	591.0 ± 2.2 ^b^	580.7 ± 0.9 ^cd^

^1^ estimated temperatures due to missing temperature sensors.

**Table 2:** Process parameters (powers, intensities, and temperatures) of the ultrasound treatments at an amplitude of 80% for 12 min. Values are means ± standard deviation of process parameters given by the ultrasound instrument every 10 s.

	Untreated	HPP	PEF	LP	CP	Hot Filling
Power (W)	26.3 ± 3.8	26.2 ± 4.3	25.3 ± 4.2	25.4 ± 4.1	25.8 ± 4.1	24.5 ± 3.8
Intensity (W/cm^2^)	68.3 ± 9.9	68.0 ± 11.2	65.7 ± 10.8	66.0 ± 10.6	66.9 ± 10.6	63.6 ± 9.9
Temperature (°C)	29.5 ± 4.4	30.7 ± 6.4	32.0 ± 5.7	30.5 ± 5.4	29.8 ± 5.3	29.7 ± 6.3

**Table 3:** Chromatographic, UV/Vis, and mass spectrometric characteristics of carotenoids from orange juice, obtained by HPLC-DAD-APCI- MS*^n^*.

Peak	Compound	t_R_*^a^* (min)	λ_max_ (nm)	%III/II	[M+H]^+^ (*m/z*)	HPLC/APCI (+)-MS*^n^* (Relative Intensity)
1a	(all-*E*)-auroxanthin	3.86	380, 404, 424	100	601	
1	(*Z*)-mutatoxanthin isomer I	4.50	404, 426, 453	11	585	
1b	(*Z*)-mutatoxanthin isomer II	4.52	404, 426, 453	11	585	
1c	n.i. ^b^	4.87	406, 424, 541	20	nd ^c,d^	
2	(all-*E*)-mutatoxanthin	5.27	407sh ^e^, 430, 453	15	585	MS^2^ [585]: 567 (100), 549 (20), 529 (10), 493 (13), 425(10), 377 (16), 221 (22) MS^3^ [585→567]: 549 (100),487 (10), 455 (10), 411 (12), 377 (12), 237 (11), 203 (12)
2a	apocarotenoid	5.41	426sh ^e^, 448, nd ^c^	nd ^c,f^	435	MS^2^ [435]: 417 (8), 407 (90), 393 (59), 379 (100), 323 (76), 305 (38), 267 (37), 233 (32), 229 (29), 219 (35), 201 (36) MS^3^ [435→407]: 365 (10), 351 (100), 295 (21), 247 (11), 233 (22), 219 (28), 201 (50), 159 (10) MS^3^ [435→379]: 351 (8), 323 (100), 267 (32), 229 (5), 173 (9) MS^3^ [435→323]: 305 (10), 281 (11), 267 (100), 225 (8), 211 (20), 163 (5), 99 (26)
3	(all-*E*)-lutein	6.00	420, 446, 473	42	nd ^c,d^	MS^2^ [551]: 533 (100), 495 (26), 459 (10), 429 (22), 411 (22), 397 (15), 345 (14), 175 (10) MS^3^ [551→533]: 421 (100), 365 (16), 315 (5), 259 (5), 219 (6)
4	(all-*E*)-zeaxanthin	6.49	424, 449, 475	38	569	MS^2^ [569]: 551 (10), 513 (10), 476 (36), 429 (12), 415 (10), 363 (10), 209 (8) MS^3^ [569→551]: 533 (100), 495 (23), 443 (17), 429 (22), 415 (20), 413 (19), 399 (18), 397 (22), 291 (15)
5	mutatoxanthin ester	12.57	406, 424, 446	nd ^c,f^	nd ^c,d^	549
6	n.i. ^b^	15.29	402, 420, 446	63	nd ^c,d^	
7	n.i. ^b^	16.03	417, 440, 469	93	nd ^c,d^	
8	n.i. ^b^	16.76	400, 424, 444	nd ^c,f^	nd ^c,d^	
9	n.i. ^b^	17.26	392, 424, 444	nd ^c,f^	nd ^c,d^	
10	n.i. ^b^	17.65	411, 436, 463	27	nd ^c,d^	
11	n.i. ^b^	17.98	413, 435, 463	55	nd ^c,d^	
11a	n.i. ^b^	17.72	411, 437, 461	52	nd ^c,d^	
11b	n.i. ^b^	18.02	410, 435, 462	39	nd ^c,d^	
12	n.i. ^b^	18.39	401, 424, 446	21	nd ^c,d^	
13	n.i. ^b^	18.81	399, 421, 447	78	nd ^c,d^	
13a	n.i. ^b^	18.53	401, 421, 449	26	nd ^c,d^	
13b	n.i. ^b^	18.81	401, 419, 448	34	nd ^c,d^	
14	n.i. ^b^	19.11	399, 424, 447	45	nd ^c,d^	
15	luteoxanthin ester	19.46	393, 424, 448	nd ^c,f^	755	565; 547
16	violaxanthin ester	20.09	417, 441, 469	83	nd ^c,d^	565
17	(all-*E*)-zeinoxanthin	20.56	430sh ^e^, 449, 477	58	533	MS^2^ [553]: 535 (100), 497 (25), 461 (86), 429 (40), 400 (5), 399 (22), 347 (20), 285 (12) MS^3^ [553→535]: 493 (76), 479 (100), 465 (50), 425 (72), 399 (78), 369 (65), 345 (50), 269 (44), 235 (43), 187 (48), 173 (32)
18	(all-*E*)-β-cryptoxanthin	21.24	430sh ^e^, 454, 479	26	553	MS^2^ [553]: 535 (100), 497 (14), 461 (85), 429 (20), 399 (24), 400 (12), 347 (24), 209 (10), 177 (12) MS^3^ [553→535]: 479 (100), 411 (34), 397 (60), 383 (30), 371 (62), 359 (50), 277 (40), 211 (26), 223 (21), 161 (20) MS^3^ [553→413]: 357(78), 343 (100), 293 (50), 275 (72), 221 (55), 199 (60), 147 (35)
19	(all-*E*)-violaxanthin laureate (C_12:0_)	21.99	417, 439, 469	73	783	565; 547
20	(*Z*)-luteoxanthin laureate (C_12:0_)	22.24	389, 418, 445	78	783	565
21	(all-*E*)-luteoxanthin laureate (C_12:0_)	22.66	400, 418, 445	65	783	MS^2^ [783]: 765 (100), 691 (12), 583 (46), 565 (26), 547 (8), 445 (4,) 375 (5) MS^3^ [783→765]: 747 (29), 673 (33), 565 (100), 547 (18), 417 (8), 357 (10), 221 (8)
22	(9*Z*)-violaxanthin laureate (C_12:0_)	23.05	417, 439, 469	67	783	565; 547
23	violaxanthin ester	23.50	414, 436, 468	36	nd ^c,d^	565
24	violaxanthin ester	24.14	nd ^c^, 433, 464	46	nd ^c,d^	565
25	(*Z*)-violaxanthin myristate (C_14:0_)	24.61	419, 439, 469	73	811	565; 547
26	(all-*E*)-violaxanthin myristate (C_14:0_)	25.13	422, 443, 468	nd ^c,f^	811	565; 547
27	(all-*E*)-luteoxanthin myristate (C_14:0_)	25.56	399, 419, 446	72	811	MS^2^ [811]: 793 (100), 719 (10), 583 (52), 565 (20), 547 (5), 431 (5), 375 (6) MS^3^ [811→793]: 775 (35), 701 (30), 565 (100), 547 (25), 443 (4), 385 (5)
28	(9*Z*)-violaxanthin myristate (C_14:0_)	26.10	413, 434, 464	32	811	MS^2^ [811]: 793 (100), 719 (13), 583 (90), 565 (25), 547 (5), 431 (8), 375 (8) MS^3^ [811→793]: 775 (50), 701 (12), 565 (100), 547 (12), 497 (5), 431 (5), 429 (10)
29	carotenoid ester ^g^	26.52	416, 444, 469	nd ^c,f^	nd ^c,d^	
30	carotenoid ester ^g^	26.84	400, 424, 446	nd ^c,f^	nd ^c,d^	
31	mutatoxanthin laureate (C_12:0_)	27.27	404, 424, 450	20	767	567; 549
(31)	(all-*E*)-violaxanthin palmitate (C_16:0_)	27.47	418, 438, 469	67	839	565; 547
32	antheraxanthin laureate (C_12:0_)	27.80	423, 443, 473	nd ^c,f^	767	567; 549
33	luteoxanthin palmitate (C_16:0_)	28.25	399, 419, 447	76	839	MS^2^ [839]: 583 (100), 565 (40), 547 (8), 393 (9) MS^3^ [839→821]: 803 (32), 729 (33), 565 (100), 547 (20), 375 (9)
(33)	luteoxanthin oleate (C_18:1_)	28.25	399, 419, 447	76	865	MS^2^ [865]: 847 (100), 829 (6), 773 (10), 583 (45), 565 (25), 547 (5), 485 (5),357 (5) MS^3^ [865→847]: 829 (32), 755 (40), 689 (8), 565 (100), 547 (10), 509 (8), 357 (10), 259 (8)
34	(9*Z*)-violaxanthin palmitate (C_16:0_)	28.73	413, 434, 464	70	839	MS^2^ [839]: 821 (82), 583 (100), 565 (20), 547 (5), 459 (5) MS^3^ [839→583]: 565 (100), 547 (12), 525 (14), 403 (18), 393 (8), 375 (20), 273 (12)
35	antheraxanthin myristate (C_14:0_)	29.31	421, 446, 469	23	795	567; 549
36	(all-*E*)-mutatoxanthin myristate (C_14:0_)	29.94	406sh ^e^, 424, 452	41	795	MS^2^ [795]: 777 (72), 567 (100), 549 (16), 511 (8), 359 (8) MS^3^ [795→567]: 549 (68), 511 (100), 432 (60), 378 (50), 359 (48), 313 (44), 273 (45), 253 (32)
37	(9*Z*)-mutatoxanthin myristate (C_14:0_)	30.40	407sh ^e^, 424, 449	33	795	MS^2^ [795]: 777 (83), 567 (100), 549 (12), 511 (10) 359 (12) MS^3^ [795→567]: 549 (100), 511 (20), 445 (25), 387 (28), 359 (28), 221 (30)
38	carotenoid ester ^g^	30.93	400, 424, 447	8	nd ^c,d^	
39	carotenoid ester ^g^	31.23	400, 426, 446	nd ^c,f^	nd ^c,d^	
39a	phytoene	31.38	277sh ^e^, 286, 297	nd ^c,f^	545	MS^2^ [545]: 489 (60), 463 (100), 421 (65), 395 (61), 353 (62), 325 (50), 297 (51),285 (60), 271 (38), 257 (34), 203 (28) MS^3^ [545→463]: 407 (40), 381 (40), 353 (50), 339 (51), 311 (90), 271 (85), 255 (60), 243 (60), 215 (100), 172 (41), 158 (42)
39b	phytofluene	31.53	330, 348, 367	77	543	MS^2^ [543]: 461 (100), 419 (70), 393 (90), 351(70), 325 (78), 283 (75), 269 (48), 257 (38) MS^3^ [543→461]: 419 (71), 351 (100), 339 (44), 283 (56), 241 (88), 187 (42)
40	antheraxanthin palmitate (C_16:0_)	31.85	422, 446, 473	57	823	567; 549
40a	(all-*E*)-ζ-carotene	31.94	379, 398, 423	65	541	MS^2^ [541]: 485 (50), 459 (94), 447 (50), 417 (75), 403 (100), 391 (94), 363 (47), 349 (96), 337 (75), 321 (40) MS^3^ [541→403]: 347 (100), 321 (54), 319 (46), 265 (92), 249 (48), 245 (35)
41	(*Z*)-mutatoxanthin palmitate (C_16:0_)	32.41	400, 424, 452	25	823	567; 549
42	(all-*E*)-mutatoxanthin palmitate (C_16:0_)	32.82	407sh ^e^, 424, 450	30	823	MS^2^ [823]: 805 (78), 711 (10), 567 (100), 549 (18), 511 (12), 359 (10) MS^3^ [823→567]: 549 (100), 511 (55), 445 (20), 418 (34), 359 (25), 299 (24), 239 (24)
43	(all-*E*)-α-carotene	33.42	409sh ^e^, 434, 469	32	537	481; 444
44	(all-*E*)-β-carotene	33.98	426sh ^e^, 449, 473	20	537	MS^2^ [537]: 481 (12), 445 (100), 413 (40), 399 (38), 361 (19), 347 (32), 335 (18), 281 (68), 269 (16), 177 (20) MS^3^ [537→445]: 430 (70), 339 (100), 308 (72), 293 (38), 267 (40), 255 (30), 225 (35), 202 (20), 173 (20)
45	carotenoid ester ^g^	34.54	375, 395, 416	60–76	nd ^c,d^	
46	carotenoid ester ^g^	34.94	399, 414, 442	41	nd ^c,d^	
47	(*Z*)-violaxanthin caprate-laureate (C_10:0_-C_12:0_)	35.43	414, 435, 465	79	937	MS^2^ [937]: 919 (36), 765 (100), 737 (91), 565 (12), 547 (15), 375 (10) MS^3^ [937→765]: 747 (40), 565 (100), 547 (34), 403 (18), 375 (11), 349 (9) MS^3^ [937→737]: 719 (30), 565 (100), 547 (26), 375 (18), 349 (7)
48	carotenoid ester ^g^	36.00	400, 418, 445	23	nd ^c,d^	
49	carotenoid ester ^g^	36.57	379, 400, 431	45	nd ^c,d^	
50	(*Z*)-luteoxanthin oleate (C_18:1_)	37.12	395, 418, 442	52	865	MS^2^ [865]: 847 (68), 767 (10), 597 (48), 583 (100), 565 (5), 547 (5), 509 (11), 491 (6), 339 (5) MS^3^ [865→583]: 565 (82), 547 (42), 527 (78), 509 (100), 491 (28), 477 (10), 441 (11), 399 (16), 385 (12), 315 (13)
51	(*Z*)-violaxanthin dilaureate (C_12:0_-C_12:0_)	37.42	413, 435, 465	79	965	MS^2^ [965]: 947 (26), 793 (18), 765 (100), 747 (10), 737 (14), 565 (6), 547 (10), 469 (2), 403 (8), 375 (58), 393 (1) MS^3^ [965→765]: 747 (28), 565 (100), 547 (24), 403 (10), 375 (12), 357 (5)
52	carotenoid ester ^g^	37.95	401, 419, 445	41	nd ^c,d^	
53	carotenoid ester ^g^	38.25	401, 419, 445	98	nd ^c,d^	
54	carotenoid ester ^g^	38.52	414, 436, 463	25	nd ^c,d^	
55	mutatoxanthin diester (presumably caprate-laureate) (C_10:0_-C_12:0_)	39.22	398, 418, 443	63	921	549
56	(*Z*)-violaxanthin laureate-myristate (C_12:0_-C_14:0_)	39.51	413, 436, 465	80	993	MS^2^ [993]: 975 (35), 793 (100), 765 (98), 747 (12), 565 (11), 547 (9) MS^3^ [993→793]: 775 (30), 565 (100), 547 (26), 375 (20) MS^3^ [993→793]: 747 (30), 565 (100), 547 (31), 375 (10)
57	(all-*E*)-zeinoxanthin laureate (C_12:0_)	40.05	424, 443, 473	47	735	535
58	(all-*E*)-β-cryptoxanthin laureate (C_12:0_)	40.44	431, 452, 474	38	735	MS^2^ [535]: 479 (78), 439 (50), 411 (83), 399 (100), 359(80), 345 (60) MS^3^ [535→399]: 384 (58), 343 (77), 301 (70), 279 (94), 265 (91)
59	(all-*E*)-violaxanthin dimyristate (C_14:0_-C_14:0_)	41.15	414, 443, 470	79	1021	MS^2^ [1021]: 821 (22), 793 (100), 765 (31), 565(10), 547 (9), 431 (5) MS^3^ [1021→793]: 775 (39), 565 (100), 547 (39), 403 (10), 375 (10)
(59)	mutatoxanthin dilaureate (C_12:0_-C_12:0_)	40.98	398, 418, 443	63	949	949
60	(9*Z*)-violaxanthin dimyristate (C_14:0_-C_14:0_)	41.48	418, 441, 470	79	1021	MS^2^ [1021]: 821 (40), 793 (100), 765 (50), 747 (8), 565 (9), 547 (10), 431 (5) MS^3^ [1021→793]: 775 (38), 565 (100), 547 (30), 497 (5), 375 (16) MS^2^ [1003]: 911 (40), 803 (20), 775 (100), 747 (20), 547 (20) MS^3^ [1003→775]: 547 (100), 529 (5), 491 (3), 425 (4), 221 (3)
61	zeinoxanthin myristate or α-cryptoxanthin myristate (C_14:0_)	42.13	424, 443, 469	34	763	671; 535
62	β-cryptoxanthin myristate (C_14:0_)	42.49	426sh ^e^, 449, 474	nd ^c,f^	763	671; 535
63	(*Z*)-violaxanthin myristate-palmitate (C_16:0_)	43.05	414, 437, 465	72	1049	MS^2^ [1049]: 1031 (38), 821 (95), 793 (100), 775 (10), 565 (12), 547 (9) MS^3^ [1049→793]: 775 (32), 565 (100), 547 (28), 403 (8), 375 (18)
(63)	mutatoxanthin laureate-myristate (C_12:0_-C_14:0_)	42.71	398, 418, 443	63	977	MS^2^ [977]: 777 (100), 749 (95), 549 (20), 531 (5), 359 (5) MS^3^ [977→777]: 549 (100), 531 (8), 427 (6), 359 (10), 333 (5), 237 (4)
64	(all-*E*)-lutein laureate (C_12:0_)	44.07	422, 445, 473	60	733	MS^2^ [733]: 533 (100), 494 (10), 477 (7), 411 (10), 343 (8) MS^3^ [733→533]: 518 (40), 465 (40), 411 (100), 357 (60), 345 (25), 305 (50)
(64)	(all-*E*)-zeinoxanthin palmitate (C_16:0_)	43.97	422, 445, 473	60	791	535; 479
65	(all-*E*)-β-cryptoxanthin palmitate (C_16:0_)	44.39	426sh ^e^, 454, 475	17	791	MS^2^ [791]: 698 (25), 535 (100), 443 (13), 411 (4) MS^3^ [791→535]: 520 (60), 479 (48), 439 (50), 411 (70), 397 (100)
66	antheraxanthin dimyristate (C_14:0_-C_14:0_)	44.71	418, 439, 466	61	1005	MS^2^ [1005]: 805 (30), 777 (100), 749 (25), 549 (12) MS^3^ [1005→777]: 549 (100), 531 (5), 413 (5), 359 (6)
(66)	(*Z*)-violaxanthin dipalmitate (C_16:0_-C_16:0_)	44.71	418, 439, 466	61	1077	MS^2^ [1077]: 1059 (15), 821 (100), 803 (10), 565 (5), 547 (4) MS^3^ [1077→821]: 803 (37), 631 (7), 565 (100), 547 (32), 375 (11), 357 (6)
(66)	(*Z*)-violaxanthin palmitate-oleate (C_16:0_-C_18:1_)	44.71	418, 439, 466	61	1103	MS^2^ [1103]: 1085 (25), 847 (100), 821 (85), 565 (11), 547 (8) MS^3^ [1103→847]: 829 (30), 565 (100), 547 (32), 389 (4), 375 (12), 357 (7)
67	(all-*E*)-lutein 3-*O*-myristate-3′-*O*-laureate(C_14:0_-C_12:0_)	45.70	422, 445, 473	67	961	761; 733; 533
68	antheraxanthin myristate-palmitate (C_16:0_)	46.26	422sh ^e^, 443, 469	34	1033	MS^2^ [1033]: 1015 (2), 873 (5), 805 (100), 777 (80), 549 (18), 531 (4), 359 (4) MS^3^ [1033→805]: 787 (10), 549 (100), 531 (8), 413 (9), 359 (10)
69	(all-*E*)-lutein 3-*O*-myristate-3′-*O*-palmitate(C_14:0_-C_16:0_)	47.18	423sh ^e^, 446, 473	64	1017	789; 761; 533
70	antheraxanthin dipalmitate (C_16:0_-C_16:0_)	47.65	423sh ^e^, 445, 469	37	1061	MS^2^ [1061]: 1043 (4), 901 (2), 833 (10), 805 (100), 777 (6), 683 (2), 615 (3), 549 (10) MS^3^ [1061→805]: 699 (8), 645 (10), 549 (100), 485 (2), 455 (8), 427 (8), 357(10)
71	(all-*E*)-lutein 3-*O*-palmitate-3′-*O*-myristate (C_16:0_-C_14:0_)	48.51	423, 446, 473	73	1017	789; 761; 533
72	(all-*E*)-zeaxanthin myristate-palmitate (C_14:0_-C_16:0_)	49.03	424, 450, 474	46	1017	789; 761; 533
73	(all-*E*)-lutein dipalmitate (C_16:0_-C_16:0_)	49.78	423sh ^e^, 446, 474	73	1045	789; 533
74	(all-*E*)-zeaxanthin dipalmitate (C_16:0_-C_16:0_)	50.26	423sh ^e^, 446, 474	47	1045	789; 533

^a^ Retention time on Accucore C30 column. ^b^ n.i., not identified. ^c^ nd, not detected. ^d^ [M+H]^+^ or fragment ions were not detected. ^e^ shoulder. ^f^ %III/II could not be calculated because of poor resolution of the UV/vis spectrum. ^g^ Carotenoid esters were identified due to saponification.

**Table 4:** Carotenoid concentrations in non-ultrasonicated orange juices. Different letters indicate significant differences (*p* ≤ 0.05). Values are means ± standard deviation (*n* = 3).

Carotenoids [µg/100 g Juice]	Without Ultrasonication					
	Untreated	HPP	PEF	LP	CP	Hot Filling
monohydroxylated xanthophylls	27.24 ± 1.83 ^ab^	29.64 ± 2.68 ^ab^	30.77 ± 0.68 ^a^	30.41 ± 2.79 ^a^	26.18 ± 0.52 ^ab^	24.37 ± 1.60 ^b^
β-cryptoxanthin	17.24 ± 0.95 ^ab^	19.55 ± 2.07 ^a^	19.59 ± 0.55 ^a^	19.84 ± 1.91 ^a^	16.94 ± 0.28 ^ab^	15.54 ± 1.14 ^b^
polyhydroxylated xanthophylls	12.73 ± 1.19 ^ab^	13.62 ± 1.75 ^ab^	14.80 ± 0.25 ^ab^	16.22 ± 2.20 ^a^	11.65 ± 0.80 ^b^	13.49 ± 1.40 ^ab^
total free xanthophylls	39.97 ± 0.98 ^ab^	43.26 ± 4.43 ^ab^	45.58 ± 0.51 ^ab^	46.63 ± 4.60 ^a^	37.83 ± 0.60 ^b^	37.86 ± 2.97 ^b^
epoxy carotenoids	478.27 ± 21.86 ^a^	459.10 ± 26.57 ^ab^	497.42 ± 1.96 ^a^	498.60 ± 52.62 ^a^	387.99 ± 6.30 ^b^	421.61 ± 23.20 ^ab^
monoepoxy carotenoids	204.74 ± 8.87 ^ab^	196.55 ± 12.14 ^ab^	215.83 ± 1.24 ^ab^	227.28 ± 24.20 ^a^	181.38 ± 3.61 ^b^	202.27 ± 12.08 ^ab^
diepoxy carotenoids	273.52 ± 12.99 ^a^	262.55 ± 14.42 ^ab^	281.59 ± 1.33 ^a^	271.32 ± 28.57 ^a^	206.60 ± 2.71 ^c^	219.34 ± 11.16 ^bc^
total carotenes	52.68 ± 2.24 ^a^	53.74 ± 5.58 ^a^	55.87 ± 1.24 ^a^	55.55 ± 6.86 ^a^	45.34 ± 1.33 ^a^	44.64 ± 3.04 ^a^
α-carotene	17.01 ± 0.74 ^a^	17.39 ± 1.47 ^a^	17.81 ± 0.29 ^a^	19.13 ± 2.44 ^a^	15.81 ± 0.36 ^a^	16.74 ± 1.02 ^a^
β-carotene	16.72 ± 1.04 ^a^	19.25 ± 2.18 ^a^	19.30 ± 0.55 ^a^	20.17 ± 2.75 ^a^	17.41 ± 0.31 ^a^	17.35 ± 1.22 ^a^
violaxanthin esters	223.91 ± 9.54 ^a^	215.19 ± 10.69 ^a^	229.17 ± 2.38 ^a^	219.25 ± 23.67 ^a^	164.14 ± 2.80 ^b^	176.97 ± 9.15 ^b^
luteoxanthin esters	49.62 ± 3.48 ^ab^	47.37 ± 3.73 ^ab^	52.42 ± 1.11 ^a^	52.07 ± 4.94 ^a^	42.46 ± 0.68 ^b^	42.37 ± 2.15 ^b^
epoxy carotenoid fatty acid esters	475.91 ± 22.05 ^a^	456.44 ± 25.78 ^ab^	494.69 ± 1.92 ^a^	494.91 ± 51.81 ^a^	385.45 ± 5.90 ^b^	418.11 ± 23.20 ^ab^
non-epoxy carotenoid fatty acid esters	217.66 ± 6.35 ^ab^	214.49 ± 14.42 ^ab^	230.32 ± 0.79 ^ab^	248.09 ± 28.41 ^a^	198.75 ± 6.40 ^b^	218.68 ± 15.99 ^ab^
violaxanthin laureate–myristate	24.60 ± 0.78 ^a^	25.18 ± 0.95 ^a^	25.68 ± 0.28 ^a^	21.20 ± 2.22 ^b^	13.33 ± 0.38 ^c^	11.87 ± 0.17 ^c^
violaxanthin dimyristate	36.01 ± 1.15 ^a^	37.71 ± 1.69 ^a^	37.23 ± 0.41 ^a^	29.37 ± 3.02 ^b^	19.23 ± 0.32 ^c^	17.50 ± 0.76 ^c^
total carotenoid fatty acid esters	731.68 ± 33.64 ^ab^	706.82 ± 42.44 ^ab^	760.33 ± 3.35 ^a^	788.26 ± 87.41 ^a^	620.98 ± 12.01 ^b^	679.39 ± 41.18 ^ab^
total carotenoids	879.74 ± 40.81 ^ab^	857.52 ± 55.42 ^ab^	922.58 ± 1.07 ^ab^	954.11 ± 103.98 ^a^	759.95 ± 14.60 ^b^	815.30 ± 50.15 ^ab^

**Table 5:** Carotenoid concentrations in ultrasonicated orange juices. Different letters indicate significant differences (*p* ≤ 0.05). Values are means ± standard deviation (*n* = 3).

Carotenoids [µg/100 g Juice]	With Ultrasonication					
	Untreated	HPP	PEF	LP	CP	Hot Filling
monohydroxylated xanthophylls	23.56 ± 0.54 ^bc^	22.25 ± 0.14 ^c^	27.82 ± 0.48 ^a^	23.74 ± 0.16 ^bc^	22.60 ± 0.38 ^c^	25.36 ± 1.23 ^b^
β-cryptoxanthin	13.67 ± 0.35 ^b^	13.02 ± 0.12 ^b^	16.66 ± 0.36 ^a^	14.02 ± 0.07 ^b^	13.53 ± 0.20 ^b^	15.64 ± 1.05 ^a^
polyhydroxylated xanthophylls	14.74 ± 1.69 ^a^	13.47 ± 1.44 ^a^	16.86 ± 1.16 ^a^	14.91 ± 2.04 ^a^	14.91 ± 0.12 ^a^	15.85 ± 1.28 ^a^
total free xanthophylls	38.30 ± 1.17 ^bc^	35.71 ± 1.31 ^c^	44.67 ± 1.58 ^a^	38.64 ± 1.92 ^bc^	37.51 ± 0.45 ^bc^	41.21 ± 1.71 ^ab^
epoxy carotenoids	469.26 ± 3.05 ^b^	440.00 ± 0.28 ^c^	508.89 ± 6.26 ^a^	424.13 ± 2.88 ^d^	397.42 ± 5.87 ^e^	421.99 ± 2.69 ^d^
monoepoxy carotenoids	207.70 ± 1.15 ^b^	192.41 ± 0.90 ^c^	227.36 ± 3.23 ^a^	198.30 ± 1.71 ^c^	192.97 ± 2.36 ^c^	205.27 ± 2.21 ^b^
diepoxy carotenoids	261.52 ± 2.76 ^b^	247.59 ± 0.65 ^c^	281.53 ± 3.17 ^a^	225.83 ± 1.21 ^d^	204.45 ± 3.59 ^f^	216.72 ± 2.37 ^e^
total carotenes	49.44 ± 0.78 ^b^	43.06 ± 0.33 ^e^	55.49 ± 1.07 ^a^	44.95 ± 0.41 ^de^	45.36 ± 0.49 ^d^	47.41 ± 0.71 ^c^
α-carotene	17.72 ± 0.40 ^b^	16.29 ± 0.18 ^d^	19.47 ± 0.22 ^a^	17.15 ± 0.06 ^bc^	16.84 ± 0.21 ^cd^	19.17 ± 0.24 ^a^
β-carotene	15.99 ± 0.19 ^b^	14.71 ± 0.11 ^c^	18.54 ± 0.22 ^a^	16.26 ± 0.20 ^b^	16.35 ± 0.15 ^b^	18.89 ± 0.39 ^a^
violaxanthin esters	212.00 ± 2.32 ^b^	202.11 ± 1.11 ^c^	226.34 ± 3.47 ^a^	181.33 ± 1.64 ^d^	162.24 ± 3.09 ^f^	173.03 ± 1.85 ^e^
luteoxanthin esters	49.57 ± 0.46 ^b^	45.48 ± 0.58 ^c^	55.20 ± 0.31 ^a^	44.50 ± 0.61 ^cd^	42.22 ± 0.62 ^d^	43.69 ± 2.09 ^cd^
epoxy carotenoid fatty acid esters	464.67 ± 3.73 ^b^	436.35 ± 0.30 ^c^	503.64 ± 6.09 ^a^	419.53 ± 2.02 ^d^	392.46 ± 5.95 ^e^	416.41 ± 3.13 ^d^
non-epoxy carotenoid fatty acid esters	229.74 ± 1.06 ^b^	213.68 ± 1.41 ^c^	251.67 ± 3.75 ^a^	228.36 ± 1.44 ^b^	226.27 ± 2.92 ^b^	244.63 ± 2.67 ^a^
violaxanthin laureate–myristate	19.95 ± 0.47 ^b^	21.11 ± 0.31 ^b^	22.80 ± 0.48 ^a^	14.93 ± 0.32 ^c^	11.24 ± 0.46 ^d^	12.29 ± 0.64 ^d^
violaxanthin dimyristate	27.71 ± 0.50 ^b^	29.60 ± 0.65 ^ab^	30.28 ± 0.89 ^a^	19.23 ± 0.41 ^c^	15.89 ± 0.28 ^d^	12.11 ± 0.84 ^e^
total carotenoid fatty acid esters	742.55 ± 3.77 ^b^	695.10 ± 1.69 ^c^	806.86 ± 10.24 ^a^	695.39 ± 3.44 ^c^	665.57 ± 9.62 ^d^	710.96 ± 2.71 ^c^
total carotenoids	855.47 ± 4.15 ^b^	797.47 ± 2.72 ^d^	938.46 ± 12.28 ^a^	805.95 ± 4.67 ^cd^	772.55 ± 10.81 ^e^	822.76 ± 3.91 ^c^

**Table 6:** Violaxanthin and luteoxanthin ester contents in the total fraction after in vitro digestion of the untreated and pasteurized juices without and with additional ultrasonication. Different letters indicate significant differences (*p* ≤ 0.05). Values are means ± standard deviation (*n* = 3).

	Total Violaxanthin Esters	Total Luteoxanthin Esters
without ultrasonication		
untreated	170.91 ± 4.22 ^a^	36.80 ± 1.76 ^a^
HPP	166.36 ± 2.98 ^a^	33.97 ± 3.67 ^ab^
PEF	165.71 ± 4.15 ^a^	37.10 ± 1.29 ^a^
LP	141.29 ± 5.35 ^b^	34.48 ± 1.18 ^ab^
CP	126.30 ± 0.88 ^c^	30.15 ± 0.22 ^b^
hot filling	132.70 ± 1.42 ^bc^	30.88 ± 0.73 ^b^
with ultrasonication		
untreated	201.62 ± 11.86 ^a^	44.76 ± 2.43 ^b^
HPP	203.11 ± 4.43 ^a^	41.58 ± 0.77 ^bc^
PEF	215.25 ± 7.28 ^a^	49.35 ± 1.34 ^a^
LP	175.16 ± 3.82 ^b^	40.38 ± 0.54 ^cd^
CP	159.84 ± 7.57 ^b^	38.27 ± 1.34 ^cd^
hot filling	163.39 ± 1.75 ^b^	37.37 ± 0.96 ^d^

## Supplementary Materials

The following are available online at https://www.mdpi.com/2076-3921/9/6/534/s1, Figure S1: Separation of carotenoids from orange juice before in vitro digestion (black) and after in vitro digestion (orange) by HPLC-DAD (450 nm). Due to slightly different weights and injection volumes, peak areas are not comparable. Peak assignment is given in Table 3.
