# Supplementary material for: Impact of Different Pasteurization Techniques and Subsequent Ultrasonication on the In Vitro Bioaccessibility of Carotenoids in Valencia Orange (Citrus sinensis (L.) Osbeck) Juice
Source: Antioxidants (Basel). 2020 Jun 18;9(6):534. doi: 10.3390/antiox9060534 (PMC7346171; doi:10.3390/antiox9060534)
Supplement: Supplementary file 1 [file antioxidants-09-00534-s001.pdf]

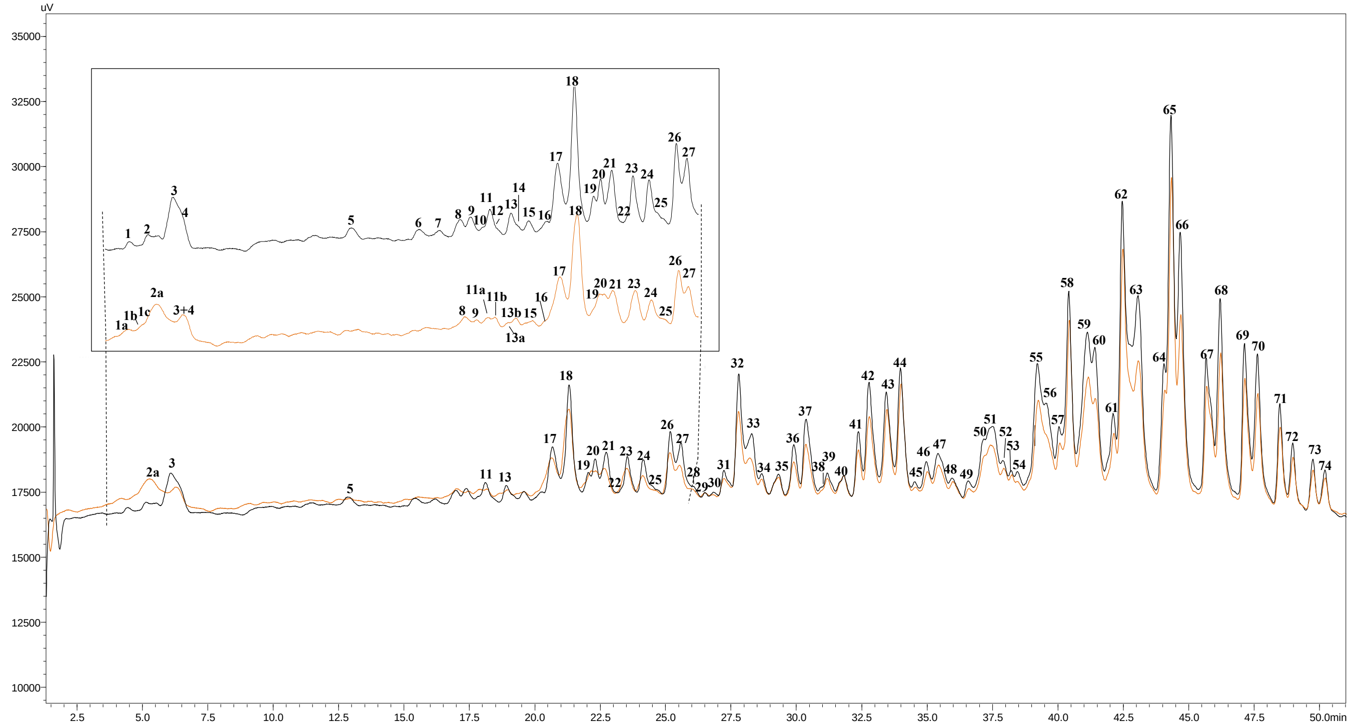

Figure S1. Separation of carotenoids from orange juice before *in vitro* digestion (**black**) and after *in vitro* digestion (**orange**) by HPLC-DAD (450 nm). Due to slightly different weights and injection volumes, peak areas are not comparable. Peak assignment is given in Table 3.
